# Coexistence of prion disease and benzodiazepine intoxication – Case report

**DOI:** 10.1186/1471-2334-14-S7-P19

**Published:** 2014-10-15

**Authors:** Radu Alexandru Macovei, Radu Ciprian Ţincu

**Affiliations:** 1 Intensive Care Unit, Clinical Emergency Hospital Bucharest, Bucharest, Romania

## Background

Human transmissible prion disease has a fatal outcome with no specific treatment. Oral benzodiazepine intoxication rarely results in significant morbidity and mortality. Thus, deep nonresponsive coma should be investigated for additional etiologies.

## Case report

A 26 year-old female presented for deliberate ingestion of 200 mg benzodiazepine associated with 500 mg metoprololum and she was admitted in our Toxicology - Intensive Care Unit one hour following the ingestion. Clinical evaluation showed a conscious patient, mildly agitated, with stable vital signs and unusual hyperosmia. She also had a history of about 10 months of psychiatric symptoms like mood disorders, memory loss and insomnia treated as depression. The interview emphasized multiple study visits in the United Kingdom and occasional intravenous abuse and substances dependence. Her urine benzodiazepine level was positive according to fluorescence polarization immunoassay method and the toxicological screening was negative for other drugs. Ten hours after ingestion she developed impressive opisthotonus and deep coma that required mechanical ventilation. Regarding the high suspicion of tetanus due to her intravenous drug administration, the infectious disease evaluation indicated anti-tetanus serum therapy. Brain tomography (CT) was in normal ranges. The cerebrospinal fluid (CSF) had a clear aspect and was normotensive, without biochemical abnormalities. After 48 hours of nonresponsive evolution a new brain CT scan identified massive cerebral edema. Brain magnetic resonance imaging (MRI) showed hyperintensities on T2 sequences in basal ganglia bilaterally along with diffusion restriction in the same areas, being suggestive of progressive multifocal leukoencephalopathy. Considering these aspects we performed PCR for the detection of JC virus, which was negative. A prion disease was highly suspected and 14-3-3 protein immunoassay on CSF was positive. After 2 months spent in the Critical Care Unit, the patient died through multiple organ dysfunctions.

## Conclusion

Our case underlines the importance of keeping an open mind when dealing with unexpected and unusual evolution of acute intoxication.

## Consent

Written informed consent was obtained from the patient’s next of kin for publication of this Case report and any accompanying images. A copy of the written consent is available for review by the Editor of this journal.

